# Combined irreducible femoral head fracture-dislocation (Pipkin Ⅳ) and ipsilateral irreducible intertrochanteric fracture: A case report

**DOI:** 10.1097/MD.0000000000045838

**Published:** 2025-11-28

**Authors:** Zehua Chen, Ruiqin Nie, Wu Zhang, Li Li, Lian Chen, Wuzhen He, Yahong Qu, Qiang Cheng, Jiejie Huang, Na Wu, Jun Jiang

**Affiliations:** a The Orthopaedics Hospital of Traditional Chinese Medicine, Zhuzhou City, China; b Guangxi University of Chinese Medicine, Nanning, China.

**Keywords:** case report, femoral head fracture-dislocation, intertrochanteric fracture, irreducible, Pipkin fractures

## Abstract

**Rationale:**

Irreducible femoral head fracture-dislocation (FHFD) with concomitant ipsilateral irreducible intertrochanteric fracture significantly increase the difficulty, which undescribed previously. We recorded a surgical strategy for the rare and high-energy injuries, and conducted a follow-up over a period of 4.5 months.

**Patient concerns:**

A 50-year-old male experienced a motor vehicle collision to the left hip. The assessment by X-ray and 3-dimensional (3D) computed tomography (CT) scan of the left hip confirmed a left posterior hip dislocation with an associated displaced infrafoveal femoral head fracture and a small posterior wall acetabular fracture (Pipkin IV), and an intertrochanteric fracture (Evans-Jensen type II). Both the FHFD and intertrochanteric fracture revealed irreducible characteristics.

**Diagnoses:**

Pipkin Ⅳ fracture combined with ipsilateral intertrochanteric fracture.

**Interventions:**

Open reduction and internal fixation was performed via the Kocher-Langenbeck approach, with absorbable screws fixation for the femoral head fracture and proximal femoral nail antirotation for the intertrochanteric fracture.

**Outcomes:**

In this case, the surgical approach and implant selection achieved satisfactory reduction and stabilization. Follow-up at 4.5 months postsurgery revealed well-healed fractures with no avascular necrosis on hip X-ray, along with good range of motion in the left hip and and the initiation of progressive weight-bearing.

**Lessons:**

For FHFDs demonstrating initial irreducibility, attempted closed reduction requires extreme caution to avoid iatrogenic injury. Furthermore, the current follow-up data suggest this management strategy may offer valuable insights for addressing the rare, complex FHFDs.

## 1. Introduction

Femoral head fractures (FHFs) are rare, high-energy injuries first documented by Birkett in 1869.^[1]^ Subsequently, Pipkin further categorized these fractures into 4 types—a classification system that remains the most widely used today.^[2]^ FHFs typically occur as part of complex trauma, often involving posterior hip dislocation, femoral neck or acetabular fractures, femoral shaft fractures, and associated soft-tissue damage to the capsule, labrum, and sciatic nerve.^[3,4]^ Because of the rarity of these fractures and the high incidence of complications, including femoral head avascular necrosis, posttraumatic osteoarthritis, and heterotopic ossification, achieving optimal postoperative outcomes with excellent hip function is still a clinical challenge.^[5]^ Successful management requires precise restoration of normal hip anatomy while minimizing complication risks.

It is indicated that FHFs occur in conjunction with 5 to 15% of all posterior hip dislocations.^[2]^ Due to the femoral head becoming mechanically locked behind the posterior acetabular wall, roughly 3% of femoral head fracture-dislocations (FHFDs) are irreducible upon initial presentation.^[4]^ Pipkin IV fractures are defined as FHF (type Ⅰ or Ⅱ) with acetabulum fracture, often the posterior wall resulted from posterior hip dislocation, accounting for approximately 31.3% of cases and demonstrate significantly worse prognoses compared to other types.^[6]^ To date, FHFDs with associated intertrochanteric fractures are scarely reported. In this study, we present a case of irreducible FHFD (Pipkin IV) with concomitant intertrochanteric fracture and detail its management.

## 2. Case presentation

**Figure 1.**
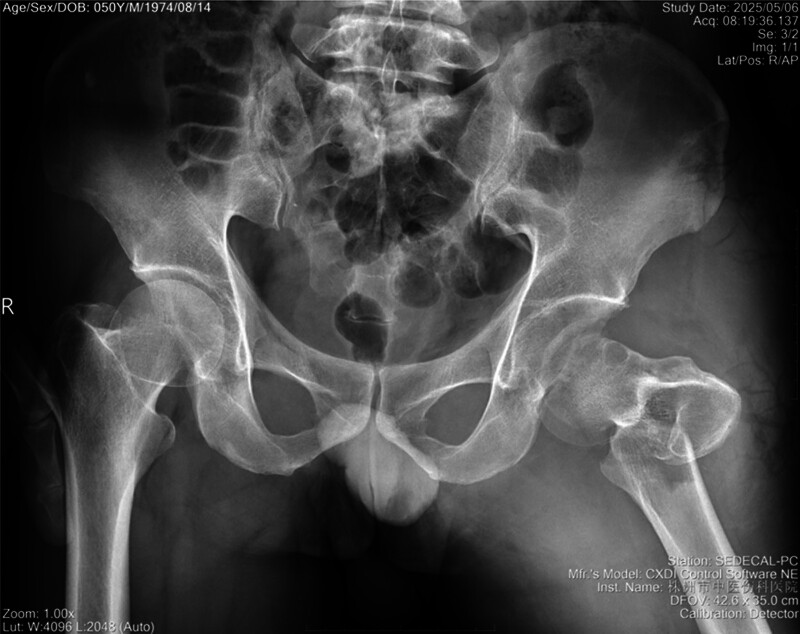
Anteroposterior pelvis radiograph.

**Figure 2.**
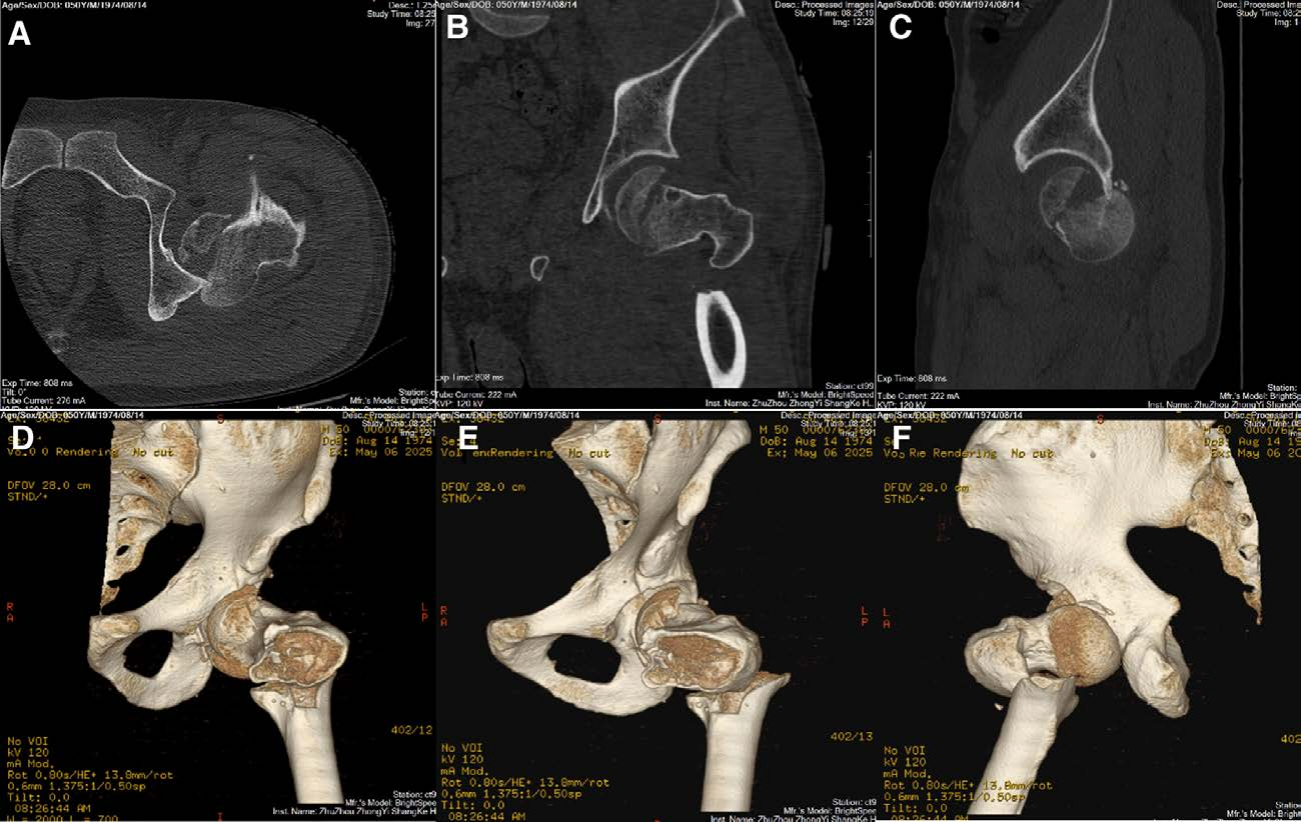
Computed tomography images in horizontal (A), coronal (B), and sagittal (C) planes along with 3D reconstruction of the left hip. A separate FHF fragment remained within the acetabulum (visible in A and C). Imaging revealed a 2-part intertrochanteric fracture with clear bisection of the lesser trochanter (visible in D, E and F).

A 50-year-old male was admitted to our hospital following a motor vehicle crash, presenting with deformity of his left lower extremity. An initial X-ray obtained in the outpatient department revealed a left posterior hip dislocation and an intertrochanteric fracture (Fig. 1). Upon hospitalization, a comprehensive evaluation was performed, including laboratory studies, chest radiography, and a 3-dimensional (3D) computed tomography (CT) scan of the left hip. The 3D CT imaging confirmed: a left posterior hip dislocation with an associated displaced infrafoveal femoral head fracture and a small posterior wall acetabular fracture (Pipkin IV), and an intertrochanteric fracture (Evans-Jensen type II) (Fig. 2). Further assessment indicated an irreducible fracture-dislocation due to the 2-part intertrochanteric fracture with bisection of the lesser trochanter, along with the femoral head lodged behind the posterior acetabular margin. Skin traction was applied with weights of 3–5 kg, and we quickly completed preoperative preparations and formulated a surgical plan.

**Figure 3.**
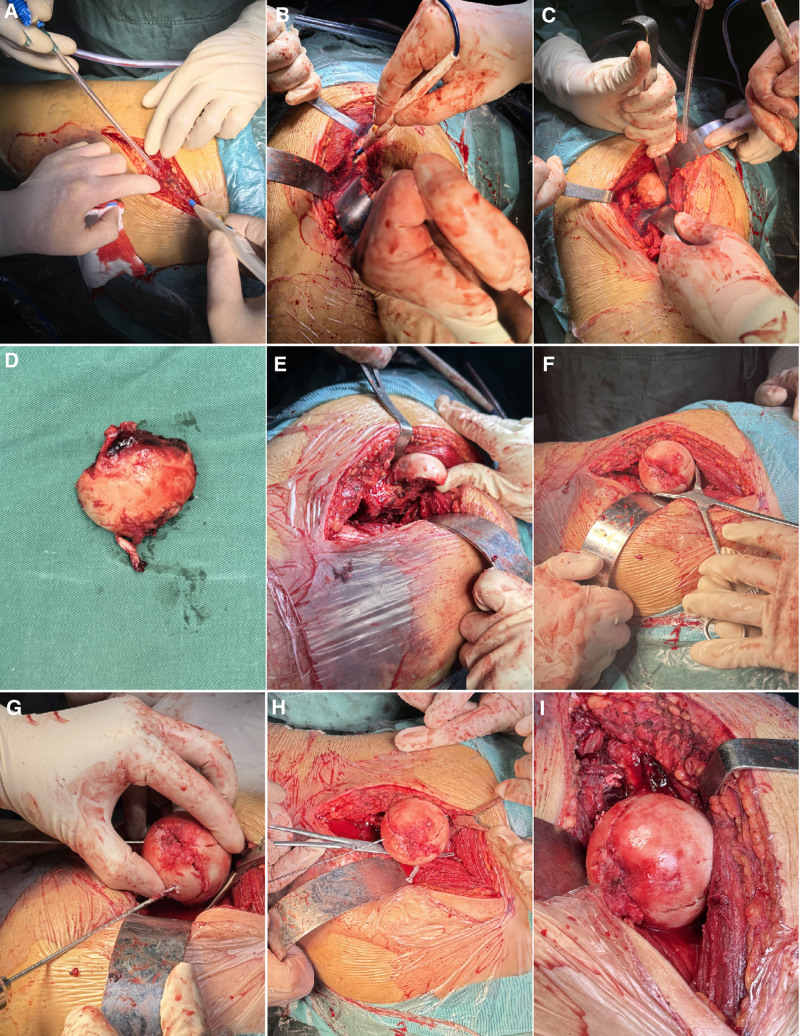
The process of surgical approach, fracture exposure, and reduction and fixation of the femoral head fracture. (A) Surgical approach. (B) Layered Incision of subcutaneous tissue. (C) Exposure of femoral head. (D) Bone fragments of femoral head. (E) Rotational exposure of femoral head fracture surface. (F) Reduction of femoral head fracture and temporary clamp fixation. (G–I) Reduction and absorbable screw fixation of femoral head fracture.

**Figure 4.**
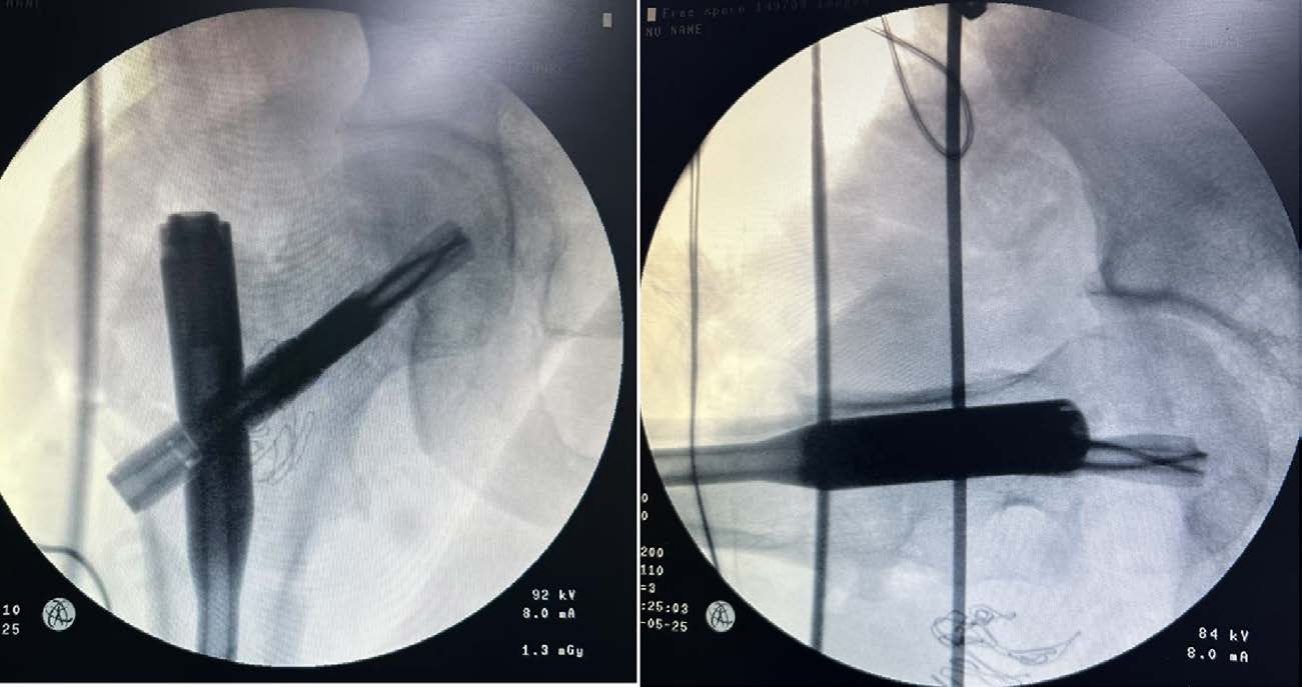
Intraoperative radiographic assessment of reduction and fixation for the intertrochanteric fracture.

**Figure 5.**
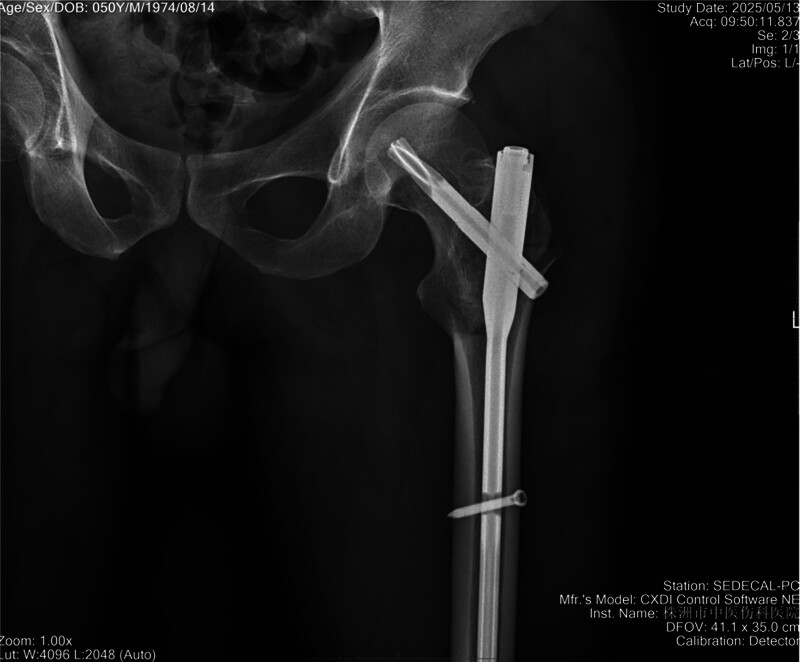
An anteroposterior radiograph of left hip was reviewed postoperatively.

An open reduction and internal fixation surgery was performed on the third day after his injury. Following successful general anesthesia and regional nerve block, the patient was placed in the right lateral decubitus position. The fracture site was adequately expose through a posterior approach (Kocker-Langenbeck, Fig. 3A). Intraoperatively, we observed that the proximal fracture segment was inverted by traction from the psoas tendon, which inserts on the superior aspect of the lesser trochanter, and the femoral head was blocked behind the posterior acetabular margin tightly. The psoas tendon and the short external rotation muscles were tenotomized, and tagged for later repair, and the femoral head was then approached (Figs. 3B and 3C). However, the femoral head remained irreducible. To facilitate reduction, the infrafoveal femoral head fragment was carefully extracted after transecting the residual tethered capsule, with meticulous attention to its preservation (Fig. 3D). To adequately visualize the fracture bed, the proximal femur was posteriorly dislocated in a internal rotation position (Fig. 3E). The infrafoveal fragment was anatomically reduced and temporarily stabilized with 0.2-mm Kirschner wires, followed by definitive fixation using two 3.7-mm absorbable Herbert screws (Fig. 3F–H). Subsequently, after reduction of the hip joint and the intertrochanteric fracture, the proximal femoral nail antirotation was inserted through the tip of the greater trochanter. The proximal femoral nail antirotation helical blade was perfectly positioned within the preexisting space created by the 2 previously placed screws (Fig. 4). After thorough irrigation of the surgical site, the small posterior wall acetabular fragment was excised. Intraoperative assessment confirmed hip joint stability, and then we sutured the wound layer by layer. During the surgery, we used autologous blood transfusion and infused 6 units of allogeneic suspension red blood cells and 200 milliliters of plasma. Postoperatively, second-generation cephalosporins were routinely administered to prevent surgical site infections, and low-molecular-weight heparin was initiated 12 hours postoperatively to reduce deep vein thrombosis risk. The drainage tube was removed when daily output decreased <50 mL. Maintain lower limb traction until 2 weeks after surgery, and nonweight-bearing for 6 weeks. We reviewed the radiographs on postoperative day 5 (Fig. 5).

## 3. Discussion

For diagnosing FHFs, while X-rays provide initial assessment, a 3D CT scan is necessary for comprehensive evaluation of the fracture and dislocation, enabling optimal treatment planning.^[3]^ To the best of our knowledge, this is the first report regarding the combination FHFD with associated ipsilateral intertrochanteric fracture. Notably, the hip dislocation and intertrochanteric fracture are irreducible. Regarding these irreducible fracture-dislocations, it is essential for surgeons to recognize that closed reduction can lead to iatrogenic femoral neck fracture,^[7]^ because the force was able to be put through the proximal femoral segment. Thus, surgical reduction is the preferred option for these fracture-dislocations. Several types of surgical approaches have been proposed, including the anterior (Smith-Petersen, Hueter), antero-lateral (Watson-Jones), lateral (Gibson) and posterior approach (Kocker-Langenbeck), among which posterior approaches are most frequently employed (52.5%), with equal utilization of surgical hip dislocation (Ganz) and the classic Kocher-Langenbeck approach.^[6]^ Certainly, each surgical approach has distinct advantages and limitations. Therefore, the optimal choice should be tailored to the specific fracture and dislocation pattern.

For isolated FHFDs, hip dislocation can typically be reduced through tracting and rotating femoral shaft. In cases of FHFDs with concomitant femoral shaft fractures, we can place a intramedullary nailing for the femoral shaft fractures stabilization, and previsionally preserve the external assist intramedullary nail device, and subsequently reduce the hip dislocation by regulating the device.^[8]^ In this case, the FHFDs is difficult to be surgically reduced because loss of intertrochanteric structural integrity eliminates the fulcrum for traction and rotation during hip dislocation reduction. Meanwhile, for the 2-part intertrochanteric femur fractures with bisection of the lesser trochanter, the upper portion of the lesser trochanter attached with the psoas tendon remained with the proximal head-neck fragment, while the lower portion attached with the ilioas tendon was a part to the distal femur shaft.^[9]^ The reduction and fixation of these fractures are also extremely challenging. Thus, We opted for Kocker-Langenbeck approach not only to reduce the blocked femoral head but also to achieve better exposure and reduction of the irreducible intertrochanteric fracture.

In this case, major surgical trauma and prolonged operation time can elevate the risk of surgical site infection. Measures such as additional intraoperative antibiotic dosing, autologous blood transfusion, and postoperative nutritional support contribute to infection prevention. Strict adherence to aseptic protocols during surgery is essential, and the use of N95 masks by operating surgeons and nurses may also be considered.^[10]^ Of course, femoral head avascular necrosis is the an undeniable complication. As reported, Ganz approach can preserves the blood supply to the femoral head and provides improved visualization.^[11]^ However, another study demonstrated similar results of standard Kocher-Langenbeck and flip osteotomy for functional outcome and complication rate.^[12]^ Additionally, total hip arthroplasty may be considered as a viable treatment option.^[6,13]^ Given the patient’s age, we opted for open reduction and internal fixation. This treatment strategy offers a high probability of intertrochanteric fracture healing, and we can preserve the option for a standard femoral stem prosthesis rather than an extended or Wagner stem when hip arthroplasty become necessary due to avascular necrosis in the future. Moreover, this approach provides an opportunity for joint preservation, potentially delaying or even avoiding the need for revision surgery. At the follow-up on September 29, 2025 (4.5 months postoperatively), a repeat hip X-ray revealed satisfactory healing of both the intertrochanteric and FHFs, with blurred fracture lines. No signs of necrosis were observed in the femoral head on the X-ray (Fig. S1, Supplemental Digital Content, https://links.lww.com/MD/Q757). Additionally, the patient exhibited a good range of motion in the left hip and had progressively begun weight-bearing exercises for the left lower limb.

Several limitations of this study should be noted. This study has several limitations. First, the evidence provided here is of a low level and serves primarily as a reference for the surgical management of this complex fracture; further investigation is required to validate the reliability of this treatment approach. Second, the study lacks long-term follow-up, and continuous monitoring of femoral head viability and hip joint function is necessary in the future. Third, adopting a Ganz osteotomy may better preserve the blood supply to the femoral head, suggesting it could be a superior surgical alternative.

## 4. Conclusion

We present a rare and irreducible FHFD with associated ipsilateral intertrochanteric fracture. For this specific type of fracture-dislocation, closed reduction should be ineffective and may potentially increase the risk of iatrogenic injury. Furthermore, current follow-up data demonstrate satisfactory results for the management of this fracture-dislocation, which may serve as a reference for treating the complex and unique FHFDs. However, long-term follow-up remains essential to observe the complications such as fracture healing and avascular necrosis of the femoral head.

## Author contributions

**Conceptualization:** Zehua Chen, Wu Zhang, Lian Chen, Jun Jiang.

**Data curation:** Zehua Chen, Yahong Qu.

**Formal analysis:** Li Li, Wuzhen He, Yahong Qu.

**Funding acquisition:** Zehua Chen.

**Investigation:** Qiang Cheng, Jiejie Huang, Na Wu.

**Methodology:** Lian Chen.

**Resources:** Li Li, Jiejie Huang, Na Wu.

**Software:** Li Li, Jiejie Huang, Na Wu.

**Supervision:** Jun Jiang.

**Validation:** Zehua Chen, Ruiqin Nie.

**Visualization:** Ruiqin Nie, Wuzhen He.

**Writing – original draft:** Zehua Chen, Ruiqin Nie, Wu Zhang.

**Writing – review & editing:** Ruiqin Nie, Wu Zhang, Qiang Cheng.

## Supplementary Material


