# Supplementary material for: Combined irreducible femoral head fracture-dislocation (Pipkin Ⅳ) and ipsilateral irreducible intertrochanteric fracture: A case report
Source: Medicine (Baltimore). 2025 Nov 28;104(48):e45838. doi: 10.1097/MD.0000000000045838 (PMC12662404; doi:10.1097/MD.0000000000045838)
Supplement: Supplementary file 1 [file medi-104-e45838-s001.pdf]

Supplementary materials

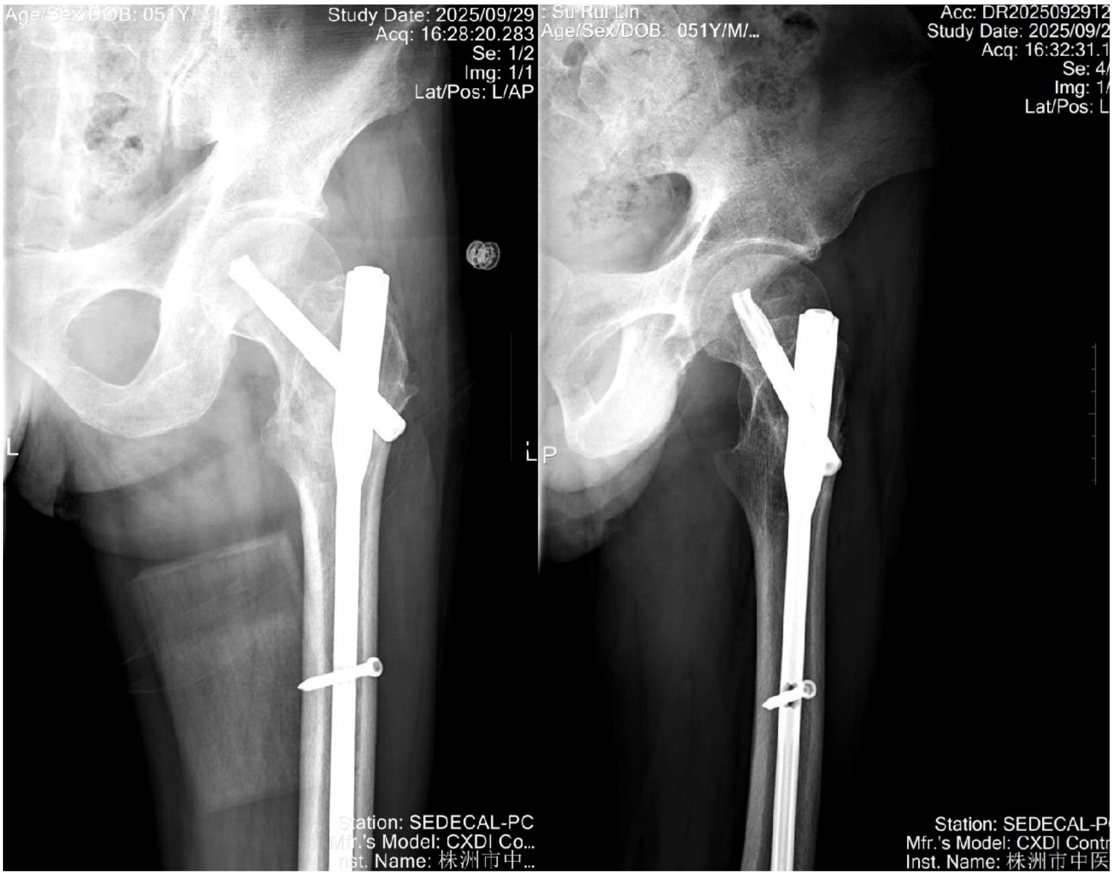

Supplementary Figure 1 A repeat X-ray of the left hip at the 4.5-month postoperative follow-up.
